# The Relation of Syphilis to General Paresis

**Published:** 1894-02

**Authors:** 


THE RELATION OF SYPHILIS TO GENERAL PARESIS.—Dr. Frederick Peterson states that his study of the subject leads him to submit the following brief conclusion:

1. A history of syphilis is found in sixty to seventy per cent. of cases of general paralysis of the insane.

2. The fact must not be lost sight of that in thirty to forty per cent. of these cases no history of syphilis, congenital or acquired, is to be found.

3. Antecedent syphilis is seven to ten times more frequent in general paralysis than in other forms of insanity.

4. Syphilis is therefore to be looked upon as a frequent but not constant factor in its production.

5. But paralytic dementia is not a form of specific disease, not a late syphilitic manifestation, nor is it a form of degeneration depending upon the syphilitic poison for its origin.

6. The relationship of syphilis to general paresis lies in the facts that it is a widespread disorder in all communities, that it weakens the constitution and vitiates the blood in many whom it infects, and that the system is thus prepared in many cases for the direct operation of the final etiology factors of general paresis, viz.: alcoholism, excessive venery, heredity, and mental overstrain and excitement.—*Ex.*

